# Reframing Self-Objectified Women’s Loneliness Experience: The Mediating Roles of Intrasexual Competition and Romantic Interpretation Bias

**DOI:** 10.1007/s10508-026-03533-y

**Published:** 2026-09-25

**Authors:** Yijia Dong, Xijing Wang

**Affiliations:** Department of Social and Behavioural Sciences, City University of Hong Kong, Kowloon, Hong Kong China

**Keywords:** Self-objectification, Loneliness, Intrasexual competition, Romantic interpretation bias, Women, Gender inequality

## Abstract

Despite the fact that women’s self-objectification is a psychological process embedded within interpersonal interactions, few studies have investigated the interpersonal consequences of self-objectification. Even though some studies linked women’s self-objectification with loneliness, an experience commonly occurring in interpersonal contexts, they produced inconsistent results regarding the association and still explained it from an intrapersonal perspective. Through the lens of interpersonal dynamics, this research attempted to reframe the relationship between self-objectification and loneliness. Specifically, two studies using distinct methods were conducted to investigate whether stronger intrasexual competition tendency when interacting with women and stronger romantic interpretation bias (RIB) when interacting with men, respectively, could explain how self-objectification among women affects loneliness. Study 1, a seven-day diary study, found that female university students experiencing strong self-objectifying feelings on a given day were more likely to engage in intrasexual competition, resulting in increased loneliness that day, even after controlling for their trait self-objectification, self-perceived attractiveness, social desirability, and demographic factors, such as relationship status. Similarly, Study 2, an experiment, revealed that women primed with high self-objectification exhibited stronger intrasexual competition tendencies and heightened feelings of loneliness, even after controlling for self-perceived attractiveness, social desirability, relationship satisfaction, and demographic factors such as relationship status. Although the promoting effect of women’s self-objectification on RIB was detected in both studies, RIB could not in turn predict reduced loneliness. Our study contributes to the literature on self-objectification by exploring its interpersonal consequences and provides directions for interventions aimed at reducing women’s loneliness.

## Introduction

Sexual objectification is a process by which women are reduced to their bodies or sexual functions for the pleasure of or consumption by others (Fredrickson & Roberts, 1997). As a result of being frequently exposed to sexual objectification, women may develop a tendency to internalize the role of objects of (male) sexual desire and adopt an observer’s perspective on their own bodies. This phenomenon is referred to as self-objectification, i.e., evaluating oneself more on the basis of physical appearance than on internal characteristics like thoughts, emotions, or personality (Fredrickson & Roberts, 1997; Roberts et al., 2018). Not surprisingly, women’s self-objectification has been found to be a key cause of a number of negative intrapersonal outcomes, such as physical and mental health risks (Roberts et al., 2018), diminished self-efficacy (Gapinski et al., 2003) and impaired cognitive performance (Winn & Cornelius, 2020). However, as a process that unfolds within interpersonal interactions (i.e., sexual objectification), comparatively few studies have examined the interpersonal consequences of women’s self-objectification.

Loneliness, commonly defined as the social pain experienced by individuals when lacking social connections, represents an important aspect of social interaction (Peplau & Perlman, 1982). Scholars have shown considerable interest in loneliness, as it is widespread among individuals across almost all age groups (Nicolaisen & Thorsen, 2014) and has been consistently linked to mental and physical health problems (e.g., Cacioppo et al., 2002; DeNiro, 1995). To understand why people feel lonely, previous studies have identified numerous self-related predictors of loneliness, such as low levels of self-esteem (Vanhalst et al., 2013), social self-efficacy (Wei et al., 2005), and self-confidence (Cheng & Furnham, 2002). Notably, Teng et al. (2019) established a conceptual link between women’s self-objectification and their experience of loneliness, highlighting that body surveillance—an indicator of self-objectification—was positively associated with body shame. In turn, body shame had an inverse relationship with self-esteem, which subsequently predicted feelings of loneliness. However, while a direct calculation of the Pearson correlation revealed a significant positive relationship between body surveillance and loneliness, this association became nonsignificant within the mediation model (i.e., when controlling for the relationship between body shame and loneliness as well as that between self-esteem and loneliness). Moreover, a recent study investigated the impact of internet usage on loneliness via self-objectification among junior high school students and found a nonsignificant relationship between self-objectification and loneliness, whether through Pearson correlation or mediation analysis (Kamila et al., 2025). These findings thereby suggest that the direct relationship between women’s self-objectification and loneliness may not be particularly robust or consistent.

Despite the findings above, we proposed that an interpersonal perspective, which has been largely overlooked by previous research, may offer deeper insights into the relationship between women’s self-objectification and loneliness. Given that both self-objectification and loneliness are rooted in social interactions, it is plausible that women’s self-objectification can affect their loneliness in different ways by altering different interpersonal dynamics. Specifically, women may have altered interaction modes with both same-sex and opposite-sex peers after objectifying themselves, influencing their loneliness levels. This study aims to explore this possibility by testing if self-objectified women may engage in “problematic” interactions both with women, manifesting as engaging in intrasexual competition, and with men, as exhibiting romantic interpretation bias (RIB). Subsequently, intrasexual competition and RIB could potentially influence women’s feelings of loneliness in different directions.

### Experiencing Increased Loneliness Levels When Interacting with Women: Intrasexual Competition as the Mechanism

First, we examined self-objectified women’s interactions with other women, which should be particularly relevant to the quality of their social networks. Despite the absence of direct evidence, previous studies have suggested that women’s body surveillance, an essential element of self-objectification, is positively correlated with their mating efforts (Niu & Zheng, 2021). Additionally, a recent study showed that self-objectified women are more inclined to derive self-worth from romantic relationships (Dong et al., 2026), which could lead to a sense of mate urgency for them (Sanchez & Broccoli, 2008). Meanwhile, women’s mating motivation is closely related to their intrasexual competition tendency (i.e., competing for access to and retention of mates), as indicated by the “ovulatory competition hypothesis,” which proposes that ovulation, a period during which women have higher estrogen levels and therefore stronger mating motivations (Bakolas & Park, 2023), amplifies their intrasexual competition tendencies (Durante et al., 2014). Empirical research has also confirmed the association between mating effort and intrasexual competition, suggesting women seeking mates are more likely to engage in competitor derogation (a typical strategy of intrasexual competition) than those without such intentions (Fisher et al., 2009). Thus, we predicted that women’s self-objectification would positively predict their intrasexual competition tendency.

An elevated level of intrasexual competition tendency, in turn, is capable of predicting women’s enhanced loneliness levels. This is likely because engaging in intrasexual competition can create strong feelings of social comparison among women (Buunk & Fisher, 2009), leading to a sense of isolation. When women perceive themselves as competing for something, it may foster an environment of rivalry rather than connection, ultimately heightening feelings of loneliness. Previous empirical evidence seems to support this link. First, participating in interpersonal competition generally could lead to feelings of loneliness (e.g., Becker et al., 2021). More specifically, Jin and Ryu (2018) found a significant correlation between women’s intrasexual competition for mates and loneliness levels. In addition, indirect evidence was provided by a study which revealed that initiating indirect aggression is associated with loneliness (Storch et al., 2004). Since indirect aggression is the most common strategy used by women during intrasexual competition (e.g., Campbell, 2004; Dong et al., 2023), it is reasonable to believe that women’s intrasexual competition can be used to predict their greater level of loneliness. In sum, we hypothesized that: Women who are self-objectified tend to have stronger intrasexual competition tendencies when interacting with other women, which further predicts a higher level of loneliness (**H1**).

### Experiencing Decreased Loneliness Levels When Interacting with Men: Romantic Interpretation Bias as the Mechanism

Additionally, we examined self-objectified women’s interactions with men, which may also contribute to the quality of their social networks. Building on the findings of Dong et al. (2026), we proposed that self-objectified women tend to perceive men as sexual agents while viewing themselves as sexual objects in cross-sex interactions. This assumption derives from the social interaction model of objectification, which suggests that patriarchal ideology in society could serve as an antecedent phase that may activate women’s sexual self-objectifying goals, thereby shaping their interactions with men (Gervais et al., 2020). Considering that self-objectified women tend to deeply internalize these ideologies, they may be more inclined to adhere to the social narrative that portrays men as sexual agents and women as sexual objects in cross-sex interactions (Gervais et al., 2020; Kahalon et al., 2018). Moreover, these women may prioritize the social category of gender over other identities when interacting with others, making it easier for them to interpret cross-sex interactions through a sexual lens. Considering the close link between sex and romance in women’s eyes (e.g., Meston & Buss, 2007), self-objectified women should be more prone to perceive female-male interactions as primarily influenced by sexual and, consequently, romantic dynamics, which can heighten their sensitivity to perceived romantic cues in such interactions. In other words, they may be more inclined to exhibit RIB, characterized by the tendency to misinterpret neutral or ambiguous cross-sex interactions as being romantic in nature (Dong et al., 2026). Therefore, we hypothesized that women’s self-objectification serves as a predictor of RIB when they interact with men.

Furthermore, we predicted that RIB might reduce women’s loneliness. First, romantic relationships are a key source of social connection for women (Cross & Madson, 1997; Gabriel & Gardner, 1999), in part because these relationships can provide women with benefits such as resources and social support. In fact, one prominent goal of romantic relationships—a common example of intimate relationships—is to provide individuals with emotional bonding and intimacy (e.g., Constant et al., 2021; Sternberg & Grajek, 1984). Thus, if a woman believes many men are romantically interested in her, she may feel less lonely, as her perceived social connection needs are fulfilled. Second, previous studies suggest that some beliefs linked to women’s self-objectification, such as the sex-is-power belief, can create illusory empowerment, making women believe that they can utilize their sexuality to achieve power (Calogero & Siegel, 2018; Wang et al., 2022). Similarly, RIB, a cognitive bias associated with women’s self-objectification (Dong et al., 2026), may also offer illusory power by making them feel connected to men around them. More specifically, this perception of widespread romantic interest could create the illusion of easy access to social connection and support, reducing feelings of loneliness. Combining the evidence above, we hypothesized that: Women who are self-objectified tend to have stronger RIB when interacting with men, which in turn predicts a lower level of loneliness (H2).

### The Present Research

In this research, we aimed to investigate whether increased intrasexual competition tendency and RIB could explain the effect of women’s self-objectification on loneliness level in different directions. In particular, we hypothesized that self-objectified women are more inclined to engage in intrasexual competition when interacting with women, thereby experiencing increased levels of loneliness (H1); they are also more likely to exhibit RIB when interacting with men, thereby experiencing decreased levels of loneliness (H2). To test these hypotheses, we conducted two studies among Chinese women, including a seven-day diary study of high ecological validity (Study 1) and an experiment (Study 2). All study procedures were approved by the Institutional Review Board of the corresponding author’s institution, and informed consent was obtained from each participant. Data, analysis scripts, and study materials can be accessed via https://osf.io/qjeh7/overview?view_only=80da0005d23d4b39b8db579e6c3755e5.

#### Sample Size Determination

The diary study (Study 1) required a minimum sample size of 100 participants who contribute data for five consecutive days, as recommended by Ohly et al. (2010). A general rule of thumb is that experiments should have at least 50 participants per condition (Faul et al., 2007). Using the standards adopted in recent research (e.g., Chen et al., 2023; Wang et al., 2023, 2024), we predetermined a sample size of 100 participants per condition for Study 2. The sensitivity power analysis (Faul et al., 2009) showed that a minimum effect size of *f* = .20 (*N* = 198) could be detected under standard criteria (α = .05, 80% power).

## Study 1

Study 1 aimed to test our hypotheses with high ecological validity by adopting a seven-day diary approach. To do so, we assessed participants’ daily self-objectifying feelings, intrasexual competition behavior, RIB tendency, and loneliness. Both self-objectifying feelings and loneliness can fluctuate from day to day (e.g., Garcia et al., 2022; Stavrova et al., 2022). Moreover, interpersonal interactions occur frequently and can vary on a daily basis, making intensive longitudinal methods, such as the daily diary approach, particularly suitable for capturing the intercorrelations between these focal variables in women’s everyday social interactions.

We specifically recruited female university students for the sample in this study, and this demographic was chosen for two key reasons. First, self-objectification is notably more prevalent among young women (e.g., Tiggemann & Lynch, 2001). Second, university students typically engage in frequent and varied interactions with peers of both sexes, facilitated by activities such as attending classes, participating in group projects, and joining clubs. These factors make female university students particularly suitable for studying the interpersonal dynamics shaped by self-objectification central to our research.

## Method

### Participants and Procedure

A total of 120 heterosexual female university students were initially recruited via online advertisements. One participant failed the attention check of the baseline questionnaire and thus was not further invited to participate in the seven-day formal study; another participant provided no response at all within seven days. In addition, four participants took more than 10 min to complete the short questionnaire (typically requiring only 2–3 min) on three or more days within the seven-day period, leading to their exclusion from the analysis due to potential low-quality responses. Therefore, 114 participants were included in the final data analysis (*M*_age_ = 21.34 years, *SD* = 1.83; 86% undergraduate; 53.5% single). Among them, 107 completed all the diaries, 6 missed one or two diaries, and 1 missed four diaries.^1^

Data were collected through Qualtrics between March 5th and March 11th, 2025. Participants completed the baseline survey on March 4th, which included trait self-objectification, together with a series of control variables, and demographic information. Participants who correctly answered the attention check question in this questionnaire received a daily questionnaire link from the next day on. They were required to complete the questionnaire once a day for seven consecutive days. Self-objectification feelings, intrasexual competition behaviors, RIB, and state loneliness were measured on a daily basis. Following Ma et al. (2025), the daily questionnaire was available from 8 to 11 pm each evening and took approximately three minutes to complete.

### Baseline Measures

*Trait Self-Objectification (Control Variable).* The Self-Objectification Questionnaire (SOQ; Noll & Fredrickson, 1998) was employed to measure trait self-objectification. Participants were asked to rank the importance of ten attributes related to physical self-concept from 0 (*least important*) to 9 (*most important*), including five appearance aspects and five competence aspects of the body. To compute SOQ scores, the sum of ranks for competence-based attributes was subtracted from those for appearance-based attributes. SOQ scores would range from − 25 to + 25, with higher scores indicating greater trait self-objectification.

*Self-Perceived Attractiveness (Control Variable).* Following Teng et al. (2022), participants were asked to indicate how much they agreed with three statements on a scale of 1 (*very unattractive/very unsatisfied*) to 9 (*very attractive/very satisfied*): “How is your physical attractiveness compared to people around you?”, “How do you rate your physical appearance?”, and “How satisfied are you with your physical appearance?” Average scores were calculated, with higher scores corresponding to higher levels of self-perceived attractiveness (α = .88).

*Social Desirability (Control Variable).* The five-item version of the Marlowe-Crowne social desirability scale (Crowne & Marlowe, 1960; Hays et al., 1989) was used (1 = *very much disagree*, 7 = *very much agree*; α = .76). Higher scores show stronger levels of social desirability.

*Demographic Information.* Participants’ age, grade, major, subjective social status (Adler et al., 2000) and relationship status (single or in a relationship) were collected. Among them, age, subjective social status, and relationship status were treated as control variables in data analysis.

### Daily Measures

*State Self-Objectification.* Following Saguy et al. (2010) and Garcia et al. (2022), two items were adopted as the daily measure of self-objectifying feelings, including “In my interactions with others today, I felt more like a body than a real person” and “In my interactions with others today, I felt as if my body and my identity were separate things.” Responses were made on a seven-point scale (1 = *strongly disagree*, 7 = *strongly agree*), with higher scores indicating stronger self-objectifying feelings. The within- and between-person omega reliabilities of state self-objectification were .53 and.93, respectively.

*Intrasexual Competition Behavior.* Adapted from a recently developed scale measuring competitive daily behavior (Arthur et al., 2024), participants were asked to indicate on a daily basis whether they engaged in behaviors that can contribute to intrasexual competition advantage through self-promotion or competitor denigration. Self-promotion behaviors include appearance enhancement (“spent time on my appearance [e. g., hair, make-up, clothes] in order to get more attention than other women”) and engaging in internal social comparison (“compared myself to other women”), while competitor denigration behaviors include gossip (“gossiped about women around me”) and internally observing negatives in others (“looked for negatives in other women”). All behaviors were measured on a five-point Likert scale (1 = *not at all*, 5 = *a great deal*), and higher average scores correspond to showing more intrasexual competition behavior on a given day. The within- and between-person omega reliabilities of intrasexual competition behavior were .54 and .92, respectively.

*RIB.* Daily RIB was measured by three items adapted from Dong et al. (2026). Participants were asked to recall their interactions with men except their partners (if any) on that day and answered the following questions: “How likely do you think it is that they consider the relationship with you to be pure friendship? (*reverse-coded*),” “To what extent do you think they might have a desire to become romantically involved with you?” and “What type of relationship do you think they desire with you?” Participants were required to give their responses on seven-point Likert scales (1 = *very much unlikely/not at all/just friends*, 7 = *very much likely/very much/romantic partners*). Higher scores reflect stronger RIB. The within- and between-person omega reliabilities of RIB were .69 and .93, respectively.

*State Loneliness.* To assess participants’ loneliness feelings at the end of each day, three items were selected from the UCLA Loneliness Scale developed by Hays and Dimatteo (1987). These items include “I feel like a member of a group of friends today (*reverse-coded*),” “I have a feeling of being left out today,” and “I feel lonely today,” and were rated on a seven-point Likert scale (1 = *strongly disagree*, 7 = *strongly agree*). Higher scores reflect higher levels of state loneliness. The within- and between-person omega reliabilities of state loneliness were .59 and .89, respectively.

### Data Analysis Strategies

Data in this study comprised a multilevel structure with daily observations (Level-1, *N* = 786) nested inside participants (Level-2, *N* = 114). All analyses were conducted using Mplus 8.3 multilevel modeling.

The first step was to construct an unconditional model (i.e., no predictors at any level of analysis) that estimated basic descriptive statistics, including mean, between- and within-person variance estimates, within-person (Level-1) correlations, and intra-class correlations (ICCs) (Nezlek, 2012; Nezlek et al., 2017). Next, we examined whether daily self-objectification could influence daily loneliness, daily intrasexual competition behavior, and daily RIB using multilevel regression models (both without and with control variables included). Our final step was to establish a within-person mediation model at Level-1 to test if the effect of self-objectification on loneliness could be explained by intrasexual competition behavior and RIB (both without and with control variables included). Specifically, in this model, daily self-objectification was the predictor, daily intrasexual competition behavior and daily RIB were the mediators, and daily loneliness was the outcome. All coefficients provided were unstandardized. Missing data were handled by Mplus using a maximum likelihood estimation that utilizes full information.

## Results and Discussion

### Descriptive Analyses

All the descriptive statistics and within-person correlations among core daily variables are presented in Table 1. The ICCs for daily measures ranged from .61 to .66, indicating that multilevel analyses should be used (Sonnentag et al., 2020).

**Table 1:** Descriptive statistics and within-person correlations for the main daily variables

	*M*	Variance_b_	Variance_w_	ICC	1	2	3	4
1 SO	2.71	1.46	0.77	.65	–			
2 ICB	2.25	0.46	0.26	.64	.10	–		
3 RIB	2.28	1.30	0.66	.66	.15	.17	–	
4 Loneliness	2.25	0.72	0.47	.61	.05	.17	.01	–

SO = self-objectification; ICB = intrasexual competition behavior; RIB = romantic interpretation bias. Variance_b_ = between-subject variances; Variance_w_ = within-subject variances; ICC = intra-class correlation. Estimated via maximum likelihood in a two-level model in Mplus

### Daily Relationships Between Self-Objectification and Loneliness, Intrasexual Competition Behaviors, and Romantic Interpretation Bias

Building multilevel regression models both without and with control variables, we separately tested whether self-objectification could predict loneliness, intrasexual competition, and RIB on a daily basis. Table 2 details specific results of the associations between all predictors (i.e., daily self-objectification and all control variables) and the three daily outcome variables. Results showed a significant positive relationship between daily self-objectification and both daily intrasexual competition^2^ and daily RIB. However, the positive relationship between daily self-objectification and daily loneliness was non-significant. These effects and their significance remained the same after controlling for participants’ trait self-objectification, self-perceived attractiveness, social desirability, and demographic factors including age, relationship status, and subjective social status. Altogether, these results revealed that women who experienced stronger self-objectifying feelings on a given day were more likely to exhibit intrasexual competition behavior and interpret the interaction with men as romantic on the same day.

**Table 2:** Associations between all predictors and daily outcome variables

	Daily outcome variables		
	Loneliness	ICB	RIB
**Core Predictor:** Daily SO	*b* = 0.04, *p* = .237	*b* = 0.06, *p* = .029	*b* = 0.13, *p* = .003
**Control Variables**			
Trait SO	*b* = 0.01, *p* = .265	*b* = 0.02, *p* < .001	*b* = 0.01, *p* = .315
SPA	*b* = −0.05, *p* = .358	*b* = 0.02, *p* = .644	*b* = 0.17, *p* = .140
SD	*b* = −0.23, *p* < .001	*b* = −0.10, *p* = .111	*b* = −0.04, *p* = .749
Age	*b* = 0.03, *p* = .305	*b* = −0.02, *p* = .460	*b* = 0.04, *p* = .463
RS	*b* = −0.10, *p* = .418	*b* = −0.06, *p* = .639	*b* = −0.22, *p* = .309
SSS	*b* = 0.04, *p* = .356	*b* = 0.02, *p* = .568	*b* = −0.05, *p* = .476

All daily variables are within-person level (Level-1) variables, while all control variables are between-person level (Level-2) variables. ICB = intrasexual competition behavior; SO = self-objectification; SPA = self-perceived attractiveness; SD = social desirability; RS = relationship status; SSS = subjective social status

### Within-Person Level Mediation Model

**Fig. 1:**
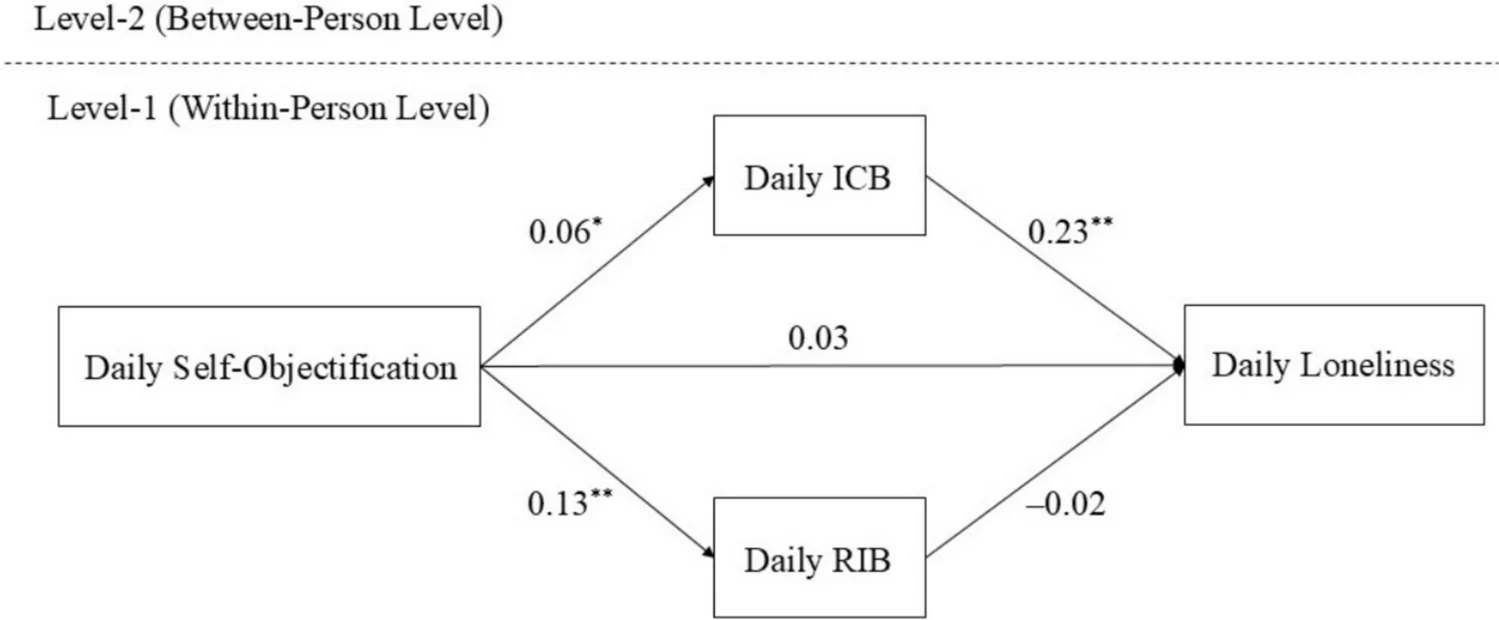
The within-person mediation model (without control variables) in Study 1. ICB = intrasexual competition behavior. ^*^p < .05, ^**^p < .01

Multilevel structural equation models were further developed to investigate the potential mediating effects of intrasexual competition and RIB for the link between women’s self-objectification and loneliness. As shown in Fig. 1, when no control variables were included, daily self-objectification had a positive effect on daily intrasexual competition (*b* = 0.06, *p* = .034), and daily intrasexual competition had a positive effect on daily loneliness (*b* = 0.23, *p* = .003). Moreover, daily intrasexual competition had a marginally significant mediation effect on the relationship between daily self-objectification and daily loneliness (*b* = 0.01, *p* = .084, 90% CI = [0.001, 0.024]).

Unexpectedly, although daily self-objectification had a significant positive effect on daily RIB (*b* = 0.13, *p* = .003), there was no significant association between daily RIB and daily loneliness (*b* = −0.02, *p* = .668). In addition, daily RIB had no significant mediation effect on the relationship between daily self-objectification and daily loneliness (*b* = −0.003, *p* = .669, 90% CI = [−0.013, 0.008]). When controlling for all the above-mentioned variables, both mediation effects remained unchanged.

Study 1 showed that women who exhibited strong feelings of self-objectification on a particular day engaged in more intrasexual competition and, therefore, might feel more lonely that day, supporting H1. In contrast, although a higher level of self-objectification also predicted a higher level of RIB, increased RIB did not further predict reduced loneliness. Therefore, H2 was not fully supported.

## Study 2

To complement Study 1, Study 2 used an experimental design to replicate its findings and, more importantly, to establish causality. To do so, we primed women’s state self-objectification and measured their state-level loneliness, intrasexual competition tendency, and RIB. Notably, previous studies have demonstrated that self-objectification can be experimentally heightened (see Kahalon et al., 2018, for a review). In this study, intrasexual competition was gauged differently than in Study 1, reflecting the shift from assessing behavior frequency to behavioral tendency. Additionally, RIB was measured using a picture-based approach as it was more ambiguous and less susceptible to interference (Dong et al., 2026).

## Method

### Participants and Design

A total of 200 Chinese heterosexual women were recruited through Credamo, an online survey platform in China similar to Prolific. Two participants who failed to answer the attention check question correctly were excluded from final analysis, leaving 198 participants in the final sample (*M*_age_ = 30.77 years, *SD* = 6.84). In this study, 4.5% of participants had never been involved in a romantic relationship, 13.1% had been involved in one in the past but were not currently involved, and 82.3% were currently involved. We randomly assigned participants to the self-objectification or control groups, resulting in 99 participants in each condition.

### Procedure and Measures

Following Dong et al. (2026), to manipulate state self-objectification, a passage reading along with a recalling task was utilized. In particular, participants were required to read a short passage in which they were informed that, influenced by cultures, media as well as interpersonal interactions, women sometimes internalize the gaze on their appearances and thus would have experiences like looking in the mirror and asking themselves how they look that day, or thinking about looking good rather than feeling comfortable when buying clothes. In other words, women could emphasize their physical appearances rather than inner qualities (such as their personalities). After that, they were asked to recall and write about any similar experiences they may have had. In contrast, participants in the control group were asked to first read that various places serve different functions to satisfy people’s daily needs in a short passage and then recall and write down experiences during their last visit to a functional place (e.g., bookstores, supermarkets). After the manipulation, participants responded to three items assessing their state self-objectification on a scale from 1 (*strongly disagree*) to 7 (*strongly agree*), including “Right now, I am thinking about how my body looks to others,” “Right now, I am imagining what my body looks like to others,” and “Right now, I feel that my body is what gives me value to other people” (α = .88; Cheng et al., 2024).

Then, intrasexual competition tendency was assessed by nine items (α = .90) from the Intrasexual Competition Scale (ICS) developed by Buunk and Fisher (2009), which assesses the extent to which individuals view their interactions with members of the same sex as competitive. Among these items, six belong to the inferiority frustration dimension and three belong to the superiority enjoyment dimension (Albert et al., 2022). The former focuses on feelings of frustration when intrasexual competitors are better off (α = .92; e.g., “I can’t stand it when I meet another woman who is more attractive than I am”), while the latter focuses on enjoyment of being better than intrasexual competitors (α = .78; e.g., “I want to be just a little better than other women”). All items were rated on a seven-point scale (1 = *strongly disagree*, 7 = *strongly agree*), and higher average scores show a stronger tendency to engage in intrasexual competition.

Next, RIB was assessed using the picture measure designed by Dong et al. (2026). Participants were sequentially presented with three cartoon pictures depicting different female-male interactant situations (i.e., playing tennis, chatting online, and riding a car together) in random order. As each picture was presented, the participant was asked to imagine that she was the female protagonist in the picture and was doing the activities depicted with a male companion. Each picture was accompanied by three items assessing RIB, including “How likely do you think it is that the man you see considers his relationship with you to be pure friendship? (*reverse-coded*)” (1 = *very much unlikely*, 7 = *very much likely*), “To what extent do you think the interaction between the man and you in this situation looks like a potential date?” (1 = *not at all*, 7 = *very much so*), and “In your opinion, what type of relationship do you think the man desires with you in this situation?” (1 = *just friends*, 9 = *romantic partners*). The three items showed a good degree of internal consistency in all three pictures (αs = .89). Following Dong et al. (2026), to obtain an overall RIB score, we averaged the scores on each item across different picture measures, standardized the three mean variables, and then averaged the three standardized variables. Higher scores indicate a stronger RIB.

After that, participants’ state loneliness was measured with three items (Hughes et al., 2023; Teng et al., 2019), including “To what extent do you feel a lack of companionship at the moment?,” “To what extent do you feel neglected at the moment?,” and “To what extent do you feel isolated at the moment?” Responses were given on a seven-point Likert scale (1 = *not at all*, 7 = *very much*; α = .86), with higher average scores implying greater loneliness.

Finally, participants provided answers to scales measuring self-perceived attractiveness (α = .92) and social desirability (α = .69) as in Study 1. Relationship satisfaction of those who were currently involved in a relationship was also measured in this study, with three items adopted from Sanchez and Broccoli (2008), including “Right now, I am very satisfied with my romantic relationship,” “I feel that my partner loves me and accepts me unconditionally,” and “I am confident that my partner will always love me.” Responses were made on a seven-point scale ranging from 1 (*strongly disagree*) to 7 (*strongly agree*). Scores were averaged, with higher scores reflecting greater relationship satisfaction (α = .85). Demographic information, including age, relationship status, and subjective social status, was also collected before participants were thanked and debriefed.

## Results and Discussion

The differences in key variables between the manipulation condition and the control condition are presented in Table 3. There was a significant difference in state-level self-objectification between the two conditions, showing the effectiveness of our manipulation method. Furthermore, women in the manipulation condition exhibited higher levels of both intrasexual competition tendency and RIB than those in the control condition. There was no significant difference observed between the two conditions in terms of state loneliness.

**Table 3:** Differences in key variables between the manipulation condition and the control condition

	Manipulation condition (*n* = 99)		Control condition (*n* = 99)		*t*(196)	*p*	Cohen’s *d*
	*M*	*SD*	*M*	*SD*			
State-level SO	5.51	0.95	4.02	1.52	8.27	< .001	1.18
Loneliness	2.89	1.32	2.65	1.18	1.32	.187	
ICT	4.37	1.16	3.76	0.95	4.02	< .001	0.57
RIB	0.16	0.94	–0.16	0.86	2.46	.015	0.35

SO = self-objectification; ICT = intrasexual competition tendency; RIB = romantic interpretation bias

As shown in Table 4, after controlling for participants’ self-perceived attractiveness, social desirability, relationship satisfaction, age, relationship status, and subjective social status, the effect of the manipulation on intrasexual competition tendency remained significant. However, the manipulation effect on RIB became non-significant, and that on state loneliness became marginally significant. The effects of control variables on the three outcome variables are also presented in Table 4.

**Table 4:** Effects of manipulation on three outcome variables with control variables included

	ICT			RIB			State loneliness		
	*F*(1, 181)	*p*	η_p_^2^	*F*(1, 181)	*p*	η_p_^2^	*F*(1, 181)	*p*	η_p_^2^
condition	9.20	.003	.048	2.63	.106	.014	3.07	.082	.017
**Control Variables**									
SPA	12.84	< .001	.066	2.99	.086	.016	7.82	.006	.041
SD	12.88	< .001	.066	18.25	< .001	.092	3.62	.059	.020
RSatisfaction	3.10	.080	.017	0.39	.535	.002	19.84	< .001	.099
Age	5.53	.020	.030	2.40	.123	.013	0.06	.815	.000
RStatus	2.80	.096	.015	0.59	.444	.003	12.04	< .001	.062
SSS	0.77	.383	.004	0.15	.695	.001	0.10	.747	.001

ICT = intrasexual competition tendency; SPA = self-perceived attractiveness; SD = social desirability; RSatisfaction = relationship satisfaction; RStatus = relationship status; SSS = subjective social status

**Fig. 2:**
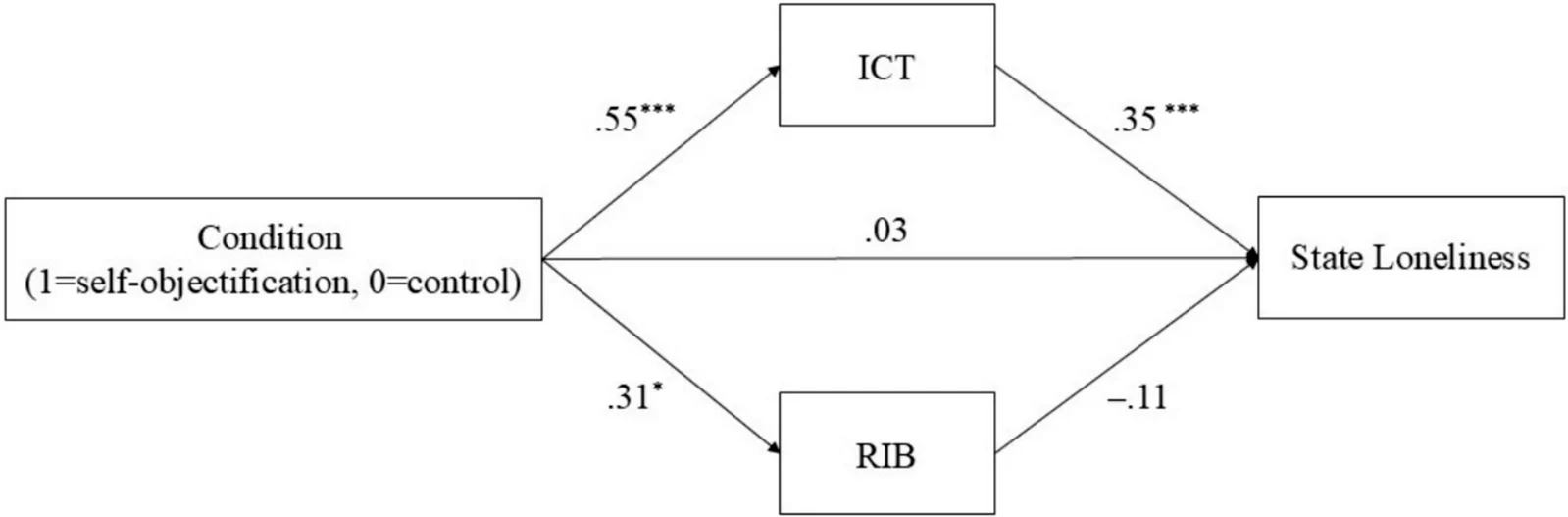
The mediation model (without control variables) in Study 2. ICT = intrasexual competition tendency. ^*^p < .05, ^***^p < .001

Using PROCESS Model 4 (a bootstrapping analysis of 5,000 random samples; Preacher & Hayes, 2008), we further examined the mediating effects of intrasexual competition tendency and RIB underlying the effect of women’s self-objectification on state loneliness both without and with control variables included. All continuous variables were standardized before being entered into the model. The results (see Fig. 2) indicated that, when no control variables were included, intrasexual competition tendency could mediate the relationship between the condition (self-objectification vs. control) and state loneliness, a*b = .19, *SE* = 0.07, 95% CI = [.074, .346]. In contrast, the mediating effect of RIB was not detected, a*b = −.03, *SE* = 0.03, 95% CI = [−.117, .020]. When including all the above-mentioned control variables in the model, the mediating effect of intrasexual competition tendency remained significant, a*b = .17, *SE* = 0.06, 95% CI = [.055, .306], while that of RIB remained non-significant, a*b = −.01, *SE* = 0.02, 95% CI = [−.048, .031].

Overall, Study 2 largely replicated the findings of Study 1. First, women primed with self-objectification were more inclined to engage in intrasexual competition, thereby reporting higher levels of state loneliness, supporting H1. However, while they were also more likely to exhibit RIB, increased RIB could not predict lower state-level loneliness, failing to fully support H2.

## General Discussion

The results of two studies involving different designs and measures support our hypothesis that self-objectified women are more likely to engage in intrasexual competition when interacting with women and thus experience higher levels of loneliness (H1). While we found a positive association between self-objectification and RIB in both studies, we were unable to show that exhibiting RIB when interacting with men by itself can predict or explain reduced loneliness levels (H2). In particular, using a daily diary design, Study 1 revealed daily positive associations between self-objectification and intrasexual competition behavior and RIB. The positive association between daily self-objectification and loneliness was found to be mediated by daily intrasexual competition behavior, even when trait self-objectification, self-perceived attractiveness, social desirability, age, relationship status, and subjective social status were controlled. Study 2 further demonstrated that self-objectification was causally related to higher levels of intrasexual competition tendency, RIB, and loneliness (with weaker evidence). In a similar manner, only intrasexual competition tendency was found to mediate the effect of self-objectification on increased loneliness, even when self-perceived attractiveness, social desirability, relationship satisfaction, age, relationship status, and subjective social status were controlled.

Importantly, in both studies, we recruited both single women and those currently in romantic relationships to enhance sample diversity and improve the generalizability of our findings. As no significant differences emerged after controlling for relationship status, this suggests that whether or not women have romantic partners, high levels of self-objectification may lead them to engage in strong intrasexual competition, resulting in greater feelings of loneliness. This aligns with previous research indicating that while relationship status can influence individuals’ strategies during intrasexual competition, it does not affect their tendency to compete (Fisher & Cox, 2011). The inclusion of this demographic diversity in our sample thus strengthens the robustness of our findings.

As noted, our research failed to identify a mediating role for RIB, primarily because we were unable to demonstrate that increased RIB among self-objectified women predicts reduced loneliness. This finding is enlightening, as it suggests that RIB may not offer any benefits to women, even when considering its potential benefiting role in cross-sex interactions. Indeed, previous research has shown that seemingly empowering beliefs held by self-objectified women—such as the sex-is-power belief—are unlikely to challenge or threaten real forms of power, including political, economic, and cultural power (Anderson, 2014). Similarly, in this study, the hypothesized soothing effect of RIB on women’s loneliness was also not observed. Thus, this research indicates that while beliefs like RIB may provide brief psychological solace or strategic thinking, it may not transform into psychological resources capable of bettering women’s actual well-being.

### Theoretical Contributions

Our research has important theoretical implications. On one hand, this study contributes to the understanding of women’s self-objectification. As mentioned, compared to the literature on intrapersonal consequences of women’s self-objectification, far less research has been conducted on its interpersonal consequences, despite the fact that self-objectification occurs primarily within interpersonal interactions (Garcia et al., 2016). Our research examines the negative interpersonal outcomes associated with self-objectification, considering both same-sex and opposite-sex relationships. Importantly, our findings offer alternative explanations for some prior studies on the intrapersonal consequences of self-objectification by adopting an interpersonal perspective. For instance, previous research has consistently linked women’s self-objectification to various physical and mental health risks (e.g., Roberts et al., 2018). Given that loneliness is a well-documented predictor of numerous mental and physical health issues (e.g., Cacioppo et al., 2002; DeNiro, 1995), it is plausible that self-objectified women are particularly vulnerable to experiencing health problems due to their maladaptive interpersonal interaction patterns, which may contribute to feelings of loneliness.

While Teng et al. (2019) focused on the potential association between women’s self-objectification and loneliness, a prevalent outcome in interpersonal processes, they focused on body shame and self-esteem as underlying mechanisms and failed to explain this association from the perspective of interpersonal dynamics. Our research has significantly broadened the scope of self-objectification literature by systematically exploring the mediating roles of intrasexual competition tendency when interacting with women and RIB when interacting with men underlying the focal association. Although our H2 was not fully demonstrated, this attempt still provides insights for illustrating previously revealed unrobust and inconsistent findings on the relationship between self-objectification and loneliness.

Finally, our study provides methodological contributions because we included self-objectification, intrasexual competition behavior and RIB in a daily diary study, examining their associations with high ecological validity. All of these variables are rooted in everyday interpersonal processes, but previous research relied heavily on cross-sectional surveys or experiments and ignored measuring their dynamics in the context of daily interactions. In particular, for the relatively new concept of RIB, although Dong et al. (2026) have proposed some intriguing measures, these are all based on imagination and are hypothetical in nature, lacking ecological validity. As a result, our study provides insights for future research in these areas, allowing them to conduct studies of a higher ecological validity.

### Practical Contributions

Individuals can suffer plenty of negative outcomes as a result of loneliness, including both mental and physical health problems (see Park et al., 2020 for a meta-analysis). It is therefore essential for researchers and practitioners to focus on the factors that influence individuals’ loneliness in order to develop effective interventions to improve people’s health and well-being. Our study identified that women’s loneliness is particularly affected by their self-objectified mindsets and problematic interactions with same-sex peers, highlighting the importance of addressing structural and systemic factors that shape women’s self-objectification as well as reducing women’s tendency to involve in intrasexual competition. For instance, programs that focus on challenging oppressive societal norms of thinness ideals of women that normalize sexual objectification can be implemented to reduce women’s self-objectification. Further, educators can assist women in viewing their relationships with same-sex peers in a more harmonious and cooperative manner and encourage them to help each other instead of engaging in continuous covert intrasexual competition. Notably, given that RIB does not alleviate women’s loneliness according to our results, it is crucial for educators or clinicians to encourage women to more accurately interpret men’s intentions and behaviors, fostering non-romance-focused cross-sex interactions.

### Limitations and Future Directions

As previously noted, we consistently found a promoting effect of self-objectification on RIB, aligning with the findings of Dong et al. (2026), but did not observe a negative association between RIB and loneliness or evidence of RIB as a mediator in the relationship between self-objectification and loneliness. These results suggest that RIB may not provide any real benefits for women, but they also open the door to other possibilities worthy of further exploration. For instance, women with strong RIB may experience greater feelings of loneliness, particularly within the context of cross-sex friendships. Research indicates that such friendships are often perceived as less competitive and more enjoyable due to complementary communication styles between genders (Holmstrom, 2009; Rawlins, 2009). For young adults, like our participants in Study 1, cross-sex friendships are crucial for social networks (Kaplan & Keys, 1997) and can serve as vital sources of social connection, potentially alleviating loneliness. However, women with RIB may doubt the likelihood of having platonic friendships with men, limiting their opportunities to form these connections, especially if they are already in romantic relationships. Additionally, the lower availability of men in women’s social networks may further contribute to these findings. Observational studies have shown that sex segregation emerges early and intensifies as children grow (Maccoby, 1988), and sociologists have noted a decline in opposite-sex peer interactions in adulthood (Kalmijn, 2002). As a result, most adult women primarily interact with female peers, which may heighten the influence of intrasexual competition on loneliness. Conversely, the limited number of men women encounter can restrict the predictive power of RIB on loneliness. Even if a self-objectified woman would exhibit heightened RIB in interactions with men, the scarcity of such opportunities may not significantly affect her loneliness. Therefore, future research should more systematically investigate RIB’s impact on women’s loneliness levels, exploring potential moderators or experimentally manipulating the number of men in women’s networks.

Additionally, while the concept of RIB offers valuable insights into the cognitive processes of self-objectified women in cross-sex interactions, it is important to acknowledge a potential limitation of this construct combined with its certain measurements, that is, it may sometimes conflate cognitive bias with actual social realities. Our Study 1 employed a daily diary approach and assessed RIB by asking participants to evaluate the likelihood of romantic intentions from men they interacted with each day. Different from the more rigorous materials depicting ambiguous cross-sex interactions used in the experimental approach, this real-time approach may inadvertently allow participants to interpret their experiences through the lens of their attractiveness, potentially influencing their reports on RIB indicators. In fact, more attractive participants may naturally receive greater romantic attention in their daily life, which could lead to higher reports on RIB measures that may reflect an accurate perception of male interest rather than a cognitive bias. Therefore, future research using such design could consider incorporating additional measures to distinguish between real romantic interest and cognitive biases, further enhancing the robustness of the RIB construct in various methodological contexts.

Finally, future studies can also investigate the consequence of men’s self-objectification from an interpersonal perspective. In the current literature, men’s self-objectification has primarily been examined in relation to their body image, drive for muscularity, or exercise behavior (Grieve & Helmick, 2008; Martins et al., 2007; Strelan & Hargreaves, 2005). It is highly likely that, similar to our findings on women’s self-objectification, men’s self-objectification can also influence their interpersonal perceptions or even behaviors, such as leading them to engage in intrasexual competition when interacting with men and exhibit sexual interpretation bias (i.e., the tendency to overestimate the sexual interest of women; Abbey, 1982) when interacting with women.

### Conclusions

Overall, the current research found that women with high levels of self-objectification tend to engage in intrasexual competition with other women, resulting in high levels of loneliness. Moreover, we replicated previous findings that women with high levels of self-objectification tend to interpret neutral or ambiguous interactions with men as romantic. However, we did not find support for the hypothesized link between increased RIB and reduced loneliness. The results of our study contribute to the literature on self-objectification and provide methodological insights. We also highlight the importance of focusing on women’s self-objectified mindset and interpersonal interactions with others (especially same-sex peers) when developing interventions aimed at reducing loneliness levels and fostering well-being among women.

## Data Availability

The data and study materials can be accessed via https://osf.io/qjeh7/overview?view_only=5d2c7435235d4f59ba0b1dab040c21e7

## Footnotes

1 In subsequent analyses, our model can adequately address the missing data.

2 As shown in Table 1, the within-level correlation between daily self-objectification and daily intrasexual competition behavior is .10, which is not very large; however, in the multilevel regression model, we found that daily self-objectification significantly predicted daily intrasexual competition behavior. This suggests that, to some extent, multilevel regression models have greater statistical power compared to traditional regression models, particularly for nested data like ours.
